# Pediatric Sinogenic Subdural Empyema: Case Report and Operative Technique

**DOI:** 10.1007/s12070-023-03754-w

**Published:** 2023-04-19

**Authors:** Giuseppe Raguso, Nicola Cornale, Rita Rebelo, Gabriele Molteni

**Affiliations:** Unit of Otorhinolaryngology, Head and Neck Department, University of Verona, Piazzale L.A. Scuro, 10, 37134 Verona, VR Italy

**Keywords:** Subdural empyema, Functional endoscopic sinus surgery, Craniotomy, Intracranial complications of sinusitis

## Abstract

Intracranial complications of sinusitis in the pediatric age are rare but potentially life threatening. They usually occur with nonspecific symptoms such as headache, fever, nausea and vomiting rather than a classic neurological presentation, but they may evolve in few hours, leading to significant morbidity with permanent brain damage and sometimes to death. For this reason, early diagnosis and prompt treatment are essential. Our case demonstrates a sinogenic subdural empyema in an immunocompetent young boy who reached our Emergency Department due to a continuous right-sided headache, unresponsive to pain relievers. The clinical history and the diagnostic process are described: at first, laboratory exams, neurologic and otolaryngological assessment were performed, together with a cranial CT scan showing an inflammatory involvement of the right frontal, ethmoidal and maxillary sinuses. Intravenous antibiotic therapy was initiated. After a few hours the patient showed a sudden worsening of his clinical conditions: he was drowsy with left lower extremity hyposthenia and ipsilateral deep tendon reflexes absence. Considering the patient’s aggravated clinical presentation an emergent MRI with contrast enhancement was conducted, showing left midline shift, a widening of the liquor space on the right frontal and parietal convexity and noticeable meningeal enhancement after contrast injection. After a Neurosurgical and ENT evaluation the patient was taken to the operating room for a combined craniotomy and trans-nasal endoscopic drainage of the empyema. We present the surgical procedure with a pictorial step-by-step description. After the surgical procedure the patient’s condition gradually improved. He regained full neurological function, was accompanied by a rehabilitation team on recovering full force on the left extremities. At discharge the patient had no apparent neurological deficits. Subdural empyema is a rare but severe complication of pediatric sinusitis. Early diagnosis with combined medical and surgical therapies play a key role to reduce morbidity and mortality.

## Introduction

Acute and chronic sinusitis is a common medical affection. In pediatric age sinusitis, if not adequately identified, can lead to complications. Severe complications, although rare, that may be associated with acute sinusitis include orbital or intracranial extension due to their contiguity to the sinuses [1].

In particular, the prevalence of intracranial complications was shown to be significantly higher in pediatric patients with frontal sinusitis [2].

Patel et al. review suggests that symptoms of sinogenic intracranial empyema in pediatric age frequently include nonspecific symptoms such as headache, fever, nausea, and vomiting.

Typical symptoms of sinusitis are barely observed, suspicion should be present when evaluating children who do not exhibit the classic neurologic presentation [3].

Our case demonstrates a subdural empyema hailed from direct extension of infection from the right frontal sinus.

## Case Report

We report a case of a previously healthy boy with a subdural empyema secondary to sinusitis.

An immunocompetent 12-year-old male adolescent was brought to the Emergency Department by his mother due to continuous headache. The boy complained of frontal, temporal right-sided pain that started 4 days earlier, that disturb the boy’s sleep and was unresponsive to pain relievers. He also reported fever with temporary resolution with paracetamol and one episode of projectile vomiting. He also referred to photophobia and decreased appetite. The patient had no history of trauma. The mother denied any chronic pathologies and his vaccination was up to date.

On the initial physical examination the patient’s vital signs were as follows: temperature 37.8 °C; heart rate 132 beats per minute, blood pressure 114/67 mmHg, respiratory rate 18 breaths per minute. The boy was ill-appearing and uncomfortable but was alert and oriented. GCS 15/15.

On neurological assessment there was no clear nuchal rigidity, but there was pain on neck flexion. Pupils were equal and reactive to light. Neurologic examination showed intact cranial nerves. Gait was uncertain due to vertigo and the Romberg test was unclear.

Motor strength showed no clear deficit. Sensation was normal. The oropharynx was erythematous, with hypertrophic tonsils. The tympanic membranes were unremarkable.

Cardiac examination revealed tachycardia without murmurs. Lungs were clear bilaterally. The abdomen was soft with normal bowel sounds.

**Fig. 1:**
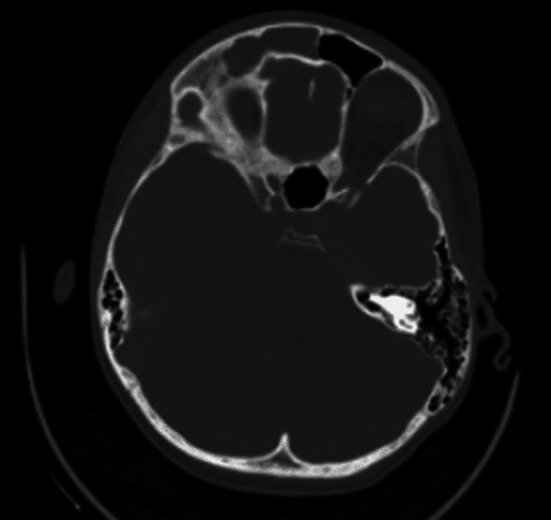
TC scan showing sinuses opacification

The laboratory results revealed: Hb 127 g/L, RBC 4.79 × 10^12^/L, WBC 15.7 × 10^9^/L, Platelets 228 × 10^9^/L, PCV 0.368 L/L, MCV 76.8 fL, MCH 26.5 pg, MCHC 345 g/L, RDW 13.6%, Neutrophils 13.51  × 10^9^/L, glucose 9.3 mmol/L, creatinine 51 μmol/L, alanine aminotransferase 21 U/L, Na 131 mmol/L, K 44.03 mmol/L, and C-reactive protein 186 mg/L. CT scan revealed modest left midline shift, no meningeal or parenchymal enhancement and inflammatory involvement of the right frontal and maxillary sinuses and right ethmoidal cells (Fig. 1).

The patient was admitted and samples were obtained for blood culture. Intravenous treatment was initiated with ceftriaxone (2 g 2 times per day) and clindamycin (600 mg 4 per day).

However on revaluation the patient was drowsy. Upper extremities strength was 5/5, sensation to touch was normal, there was no tremor, bradykinesia or clonus. When examining the lower extremities, sensation was normal, and strength was 5/5 on the right-lower extremity but left-sided extremity strength was diminished and deep tendon reflexes were absent on the left.

**Fig. 2:**
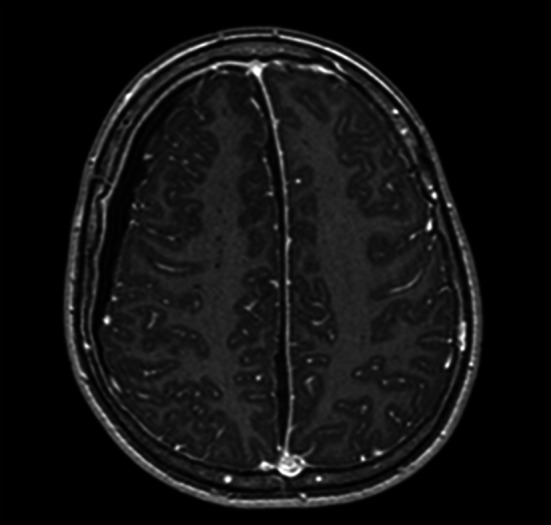
T1 sequence on axial plane demonstrating hypointense accumulation in the right hemisphere

**Fig. 3:**
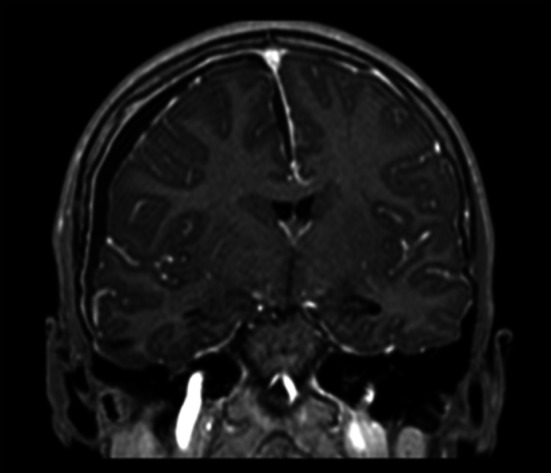
Coronal view in T1 sequence with clear liquid accumulation deviating cerebral midline

Considering the patient’s aggravated clinical presentation an emergent MRI was conducted. (Fig. 2) The liquor space widening (Fig. 3) was hyperintense in T2/FLAIR and on B1000 and ADC sequences.

**Fig. 4:**
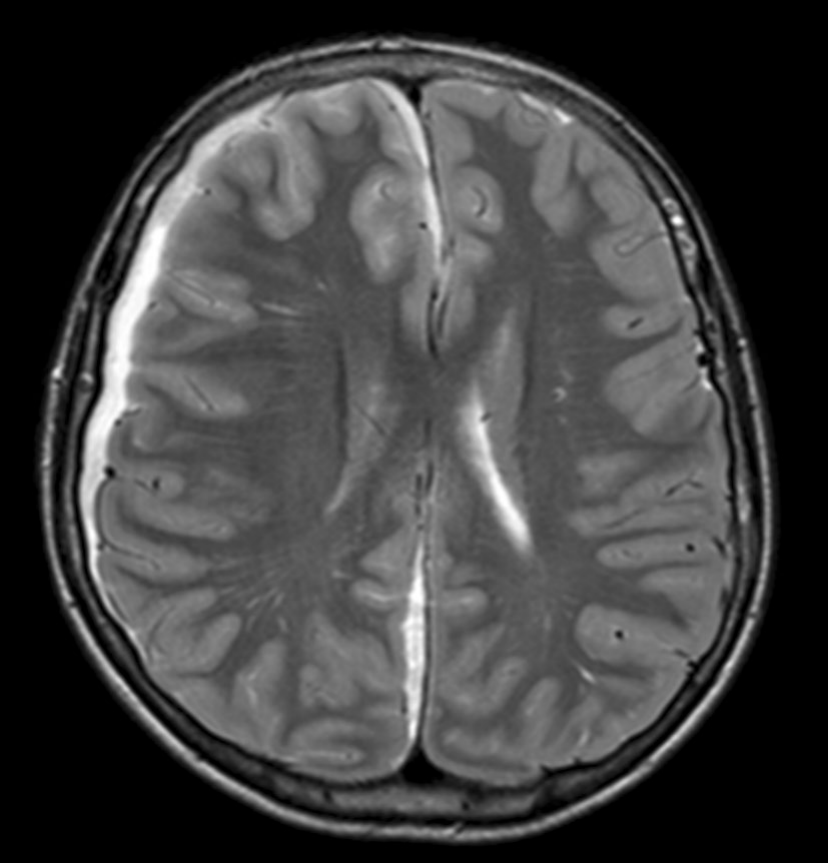
T2 sequence with hyperintensity surrounding the right hemisphere

After contrast injection there was noticeable meningeal enhancement reflecting probable inflammation (Fig. 4). It confirmed the opacification of the right nasal sinuses with sinuses wall enhancement. After a Neurosurgical and ENT evaluation the patient was taken to the operating room for craniotomy and drainage of the empyema.

**Fig. 5:**
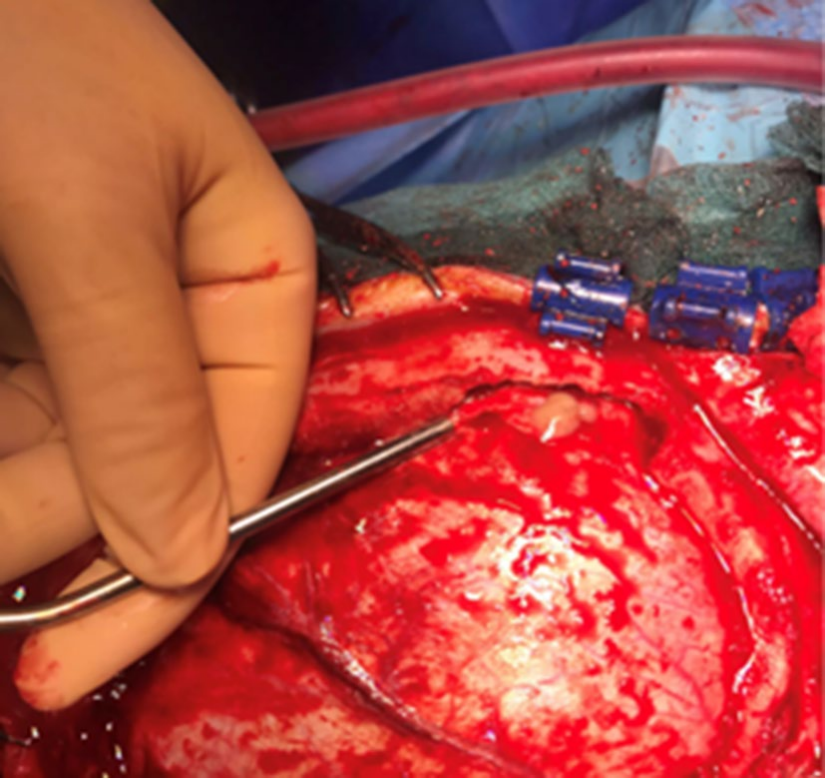
Purulent drainage from the frontal sinus leaking from frontal foramen

**Fig. 6:**
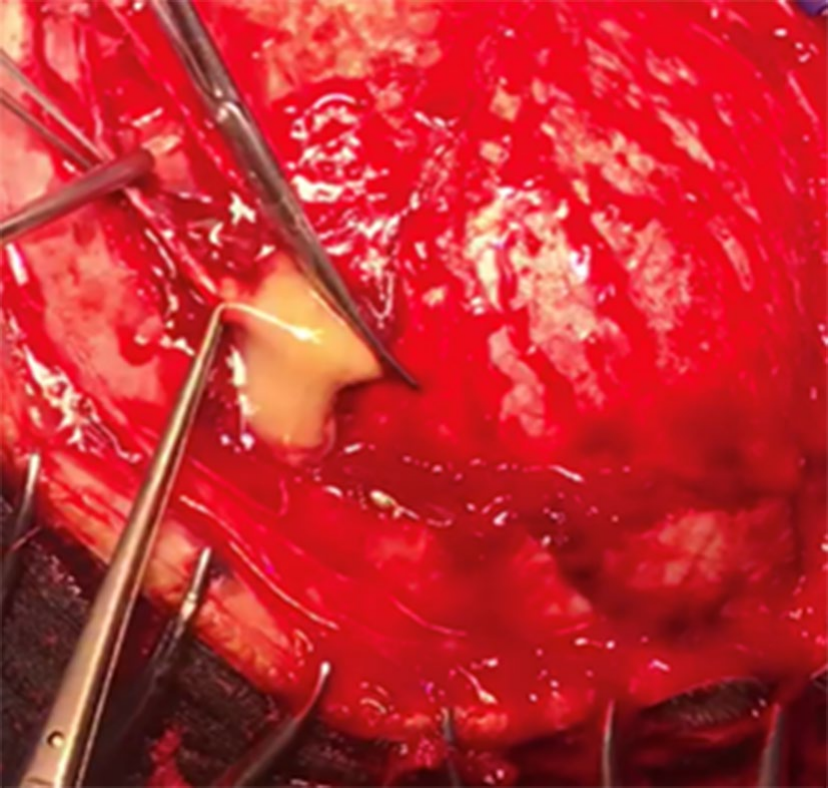
Dura mater incision showing purulent discharge in the subdural space

**Fig. 7:**
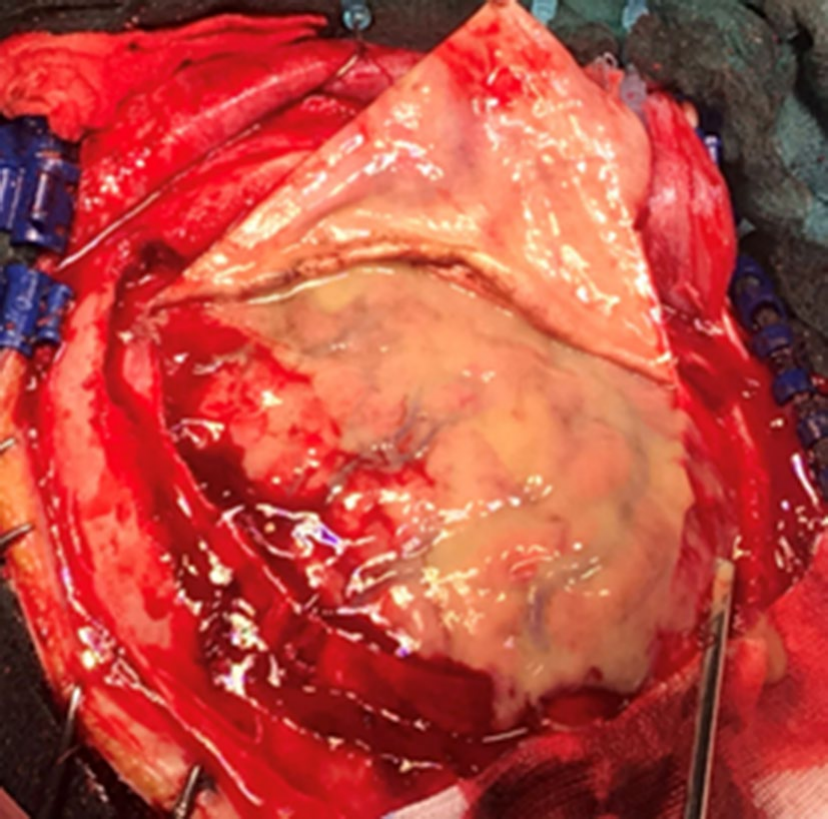
Vast purulent collection surrounded the entire hemisphere

Regarding the neurosurgical approach: Firstly, a widened right frontal, temporal parietal craniotomy was performed. Immediately after the incision it was possible to identify a purulent leakage through the frontal paramedian foramen (Fig. 5). The dura mater was then open 1 cm above the bone margin in a curvilinear manner. (Fig. 6) On opening of the membrane and exposing the brain parenchyma it was clear a vast purulent collection surrounded the entire hemisphere. (Fig. 7). The brain parenchyma was congested and the cortex hyperemic. The skull case was repositioned leaving an extra dural drainage.

**Fig. 8:**
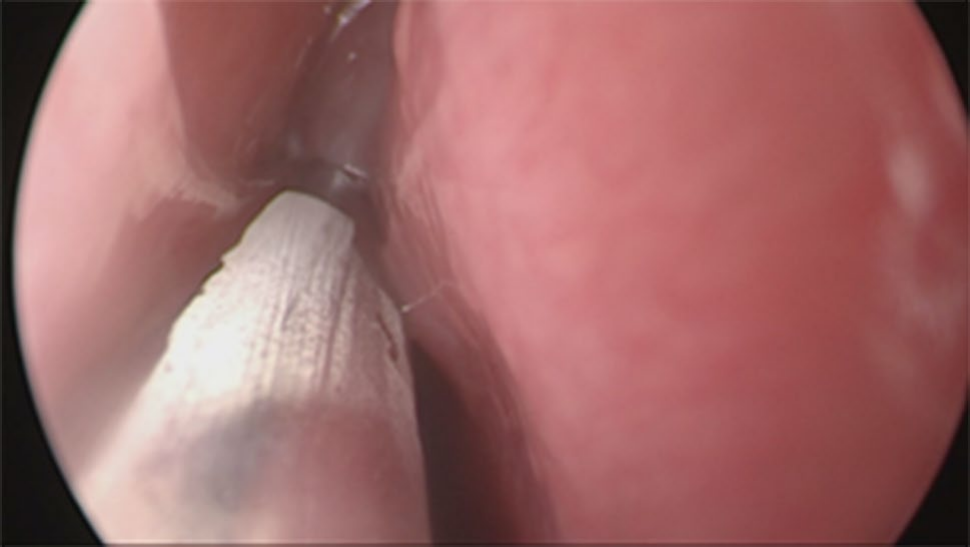
Right nasal nostril with congested mucosa

**Fig. 9:**
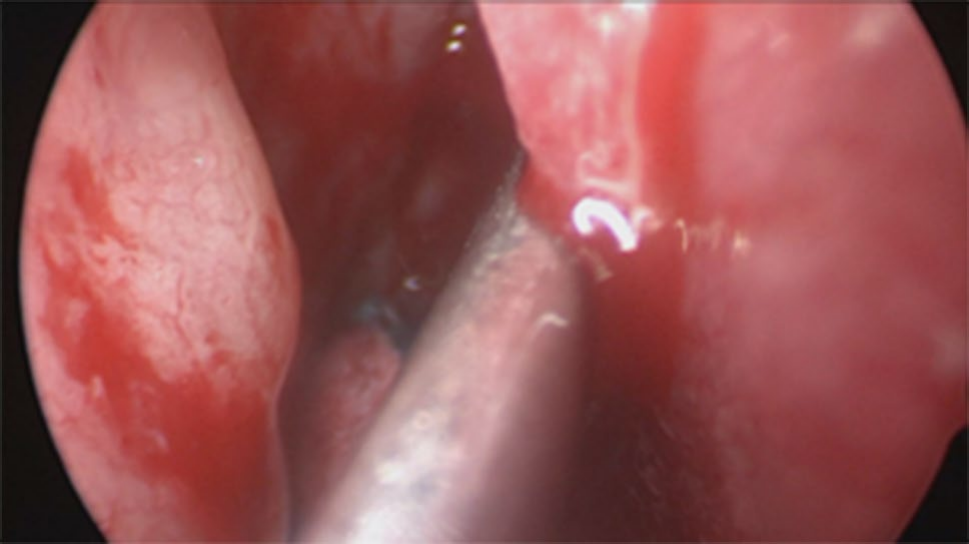
Medializing middle turbinate

**Fig. 10:**
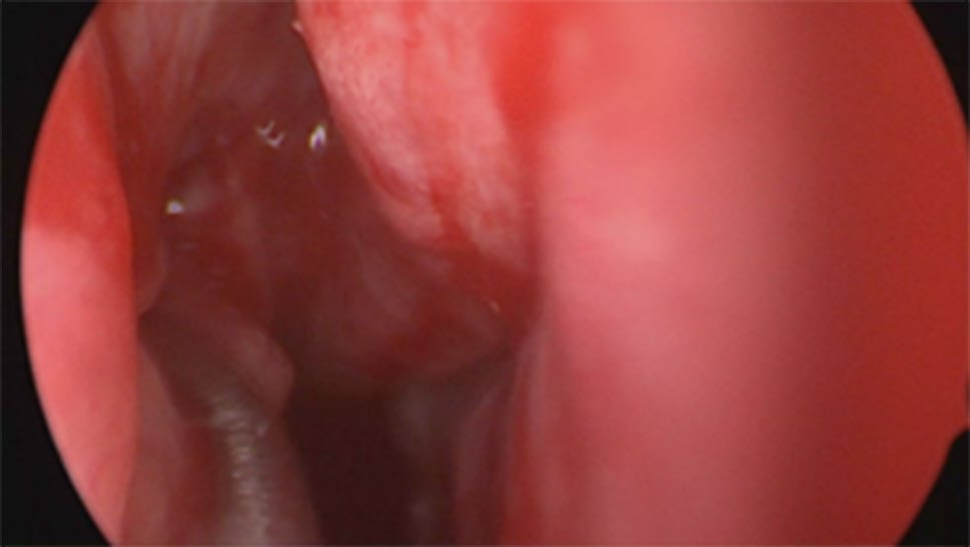
Opening of maxillary sinus

**Fig. 11:**
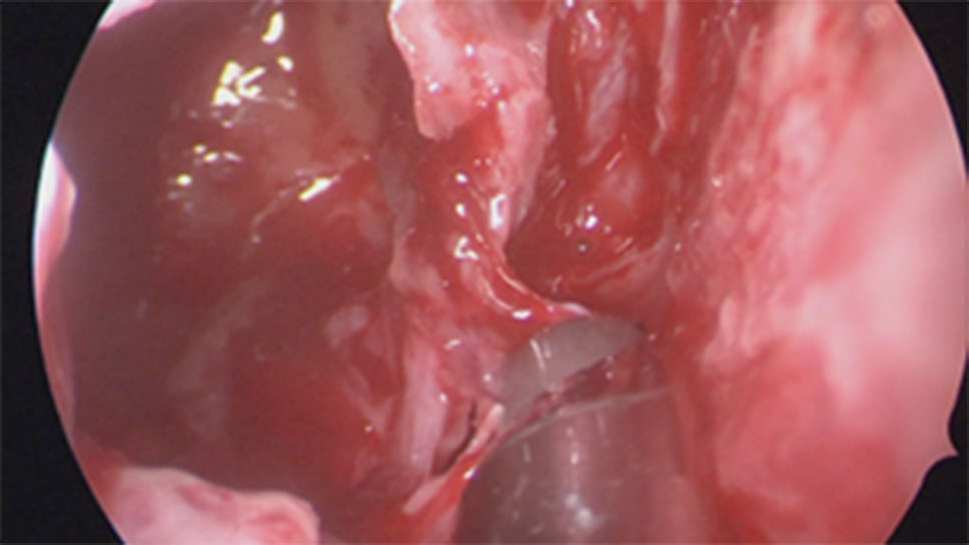
Maxillary sinus with hyperplastic mucosa, no purulent discharge

**Fig. 12:**
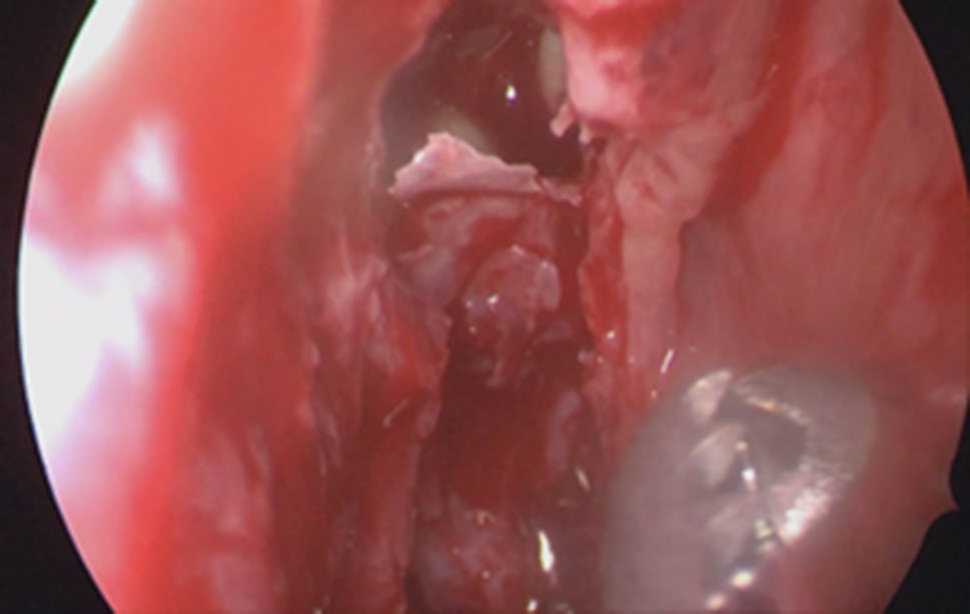
Fracture individual etmoidal septation

**Fig. 13:**
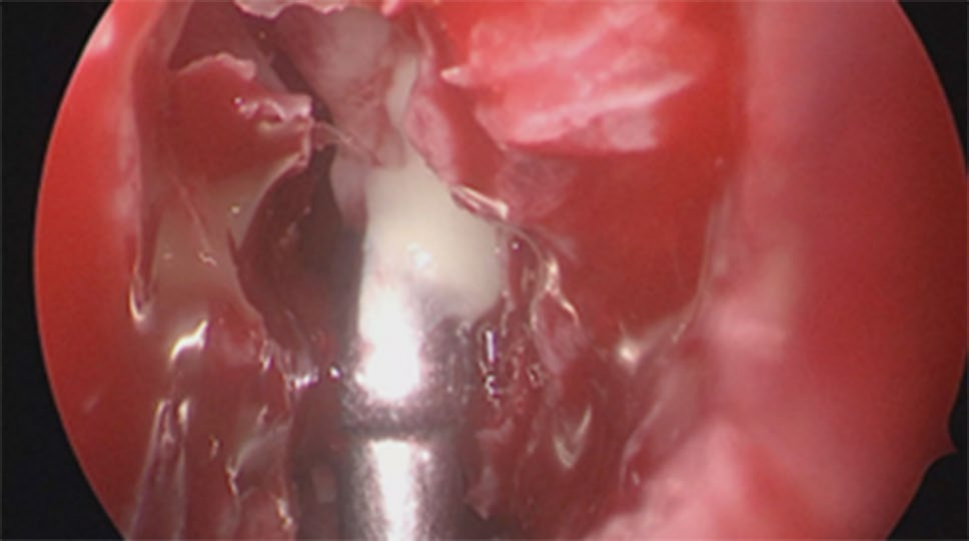
Ethmoidal cells with minimal purulent discharge

**Fig. 14:**
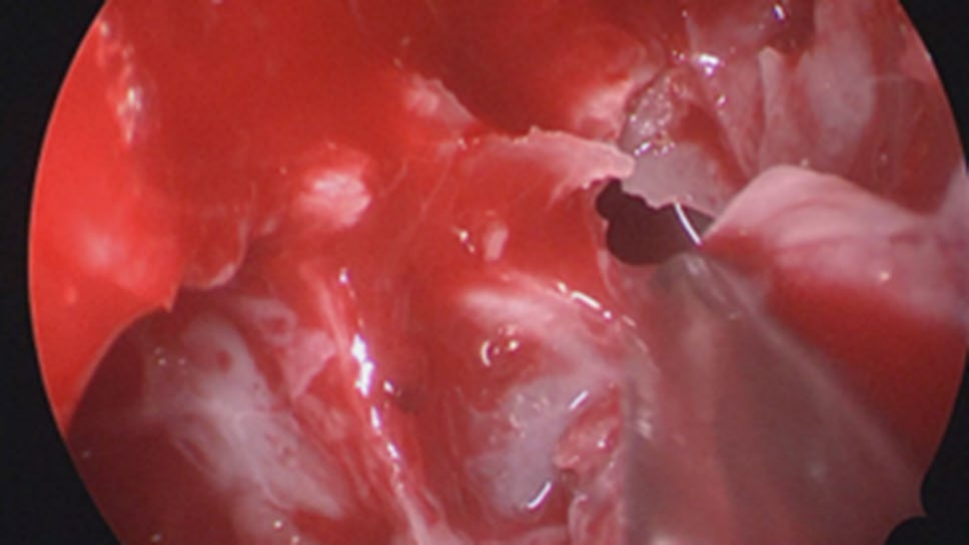
Sphenoid sinus opening

**Fig. 15:**
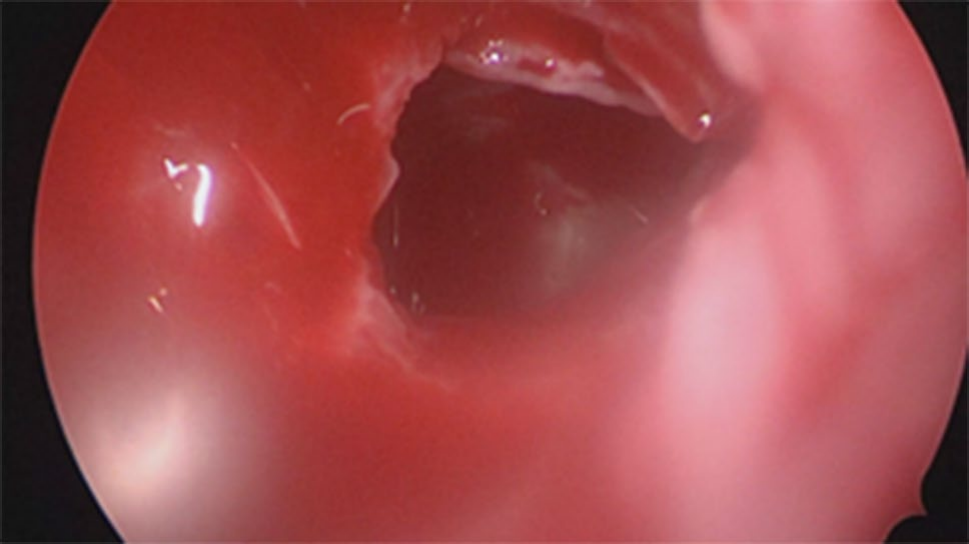
Clear sphenoid sinus

**Fig. 16:**
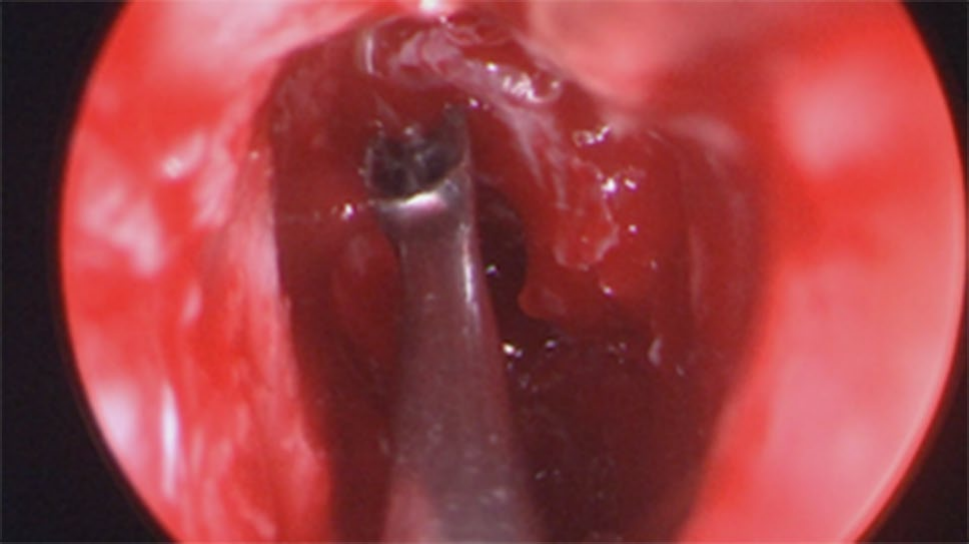
Frontal sinus identification using a curette

**Fig. 17:**
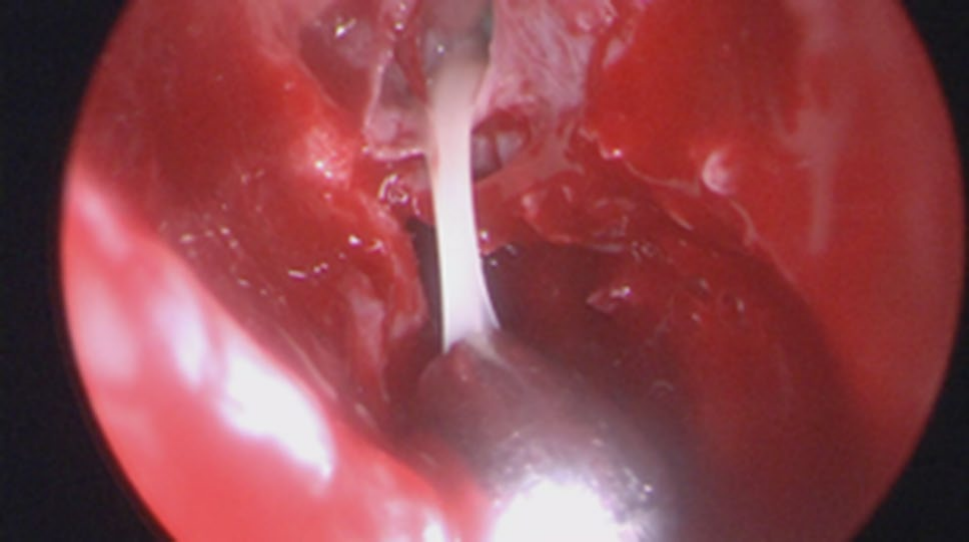
Frontal sinus suctioning of purulent discharge

**Fig. 18:**
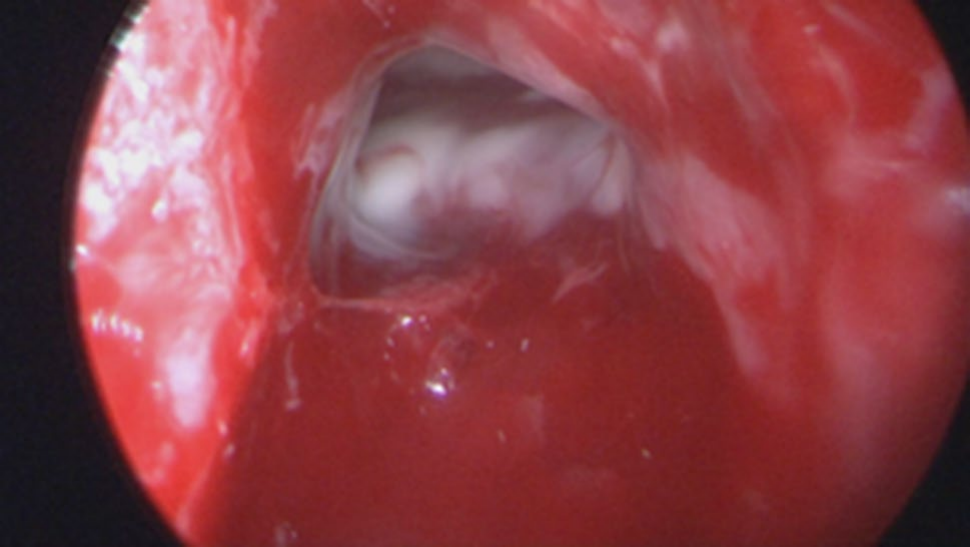
Opened frontal sinus allowing better identification of secretions

**Fig. 19:**
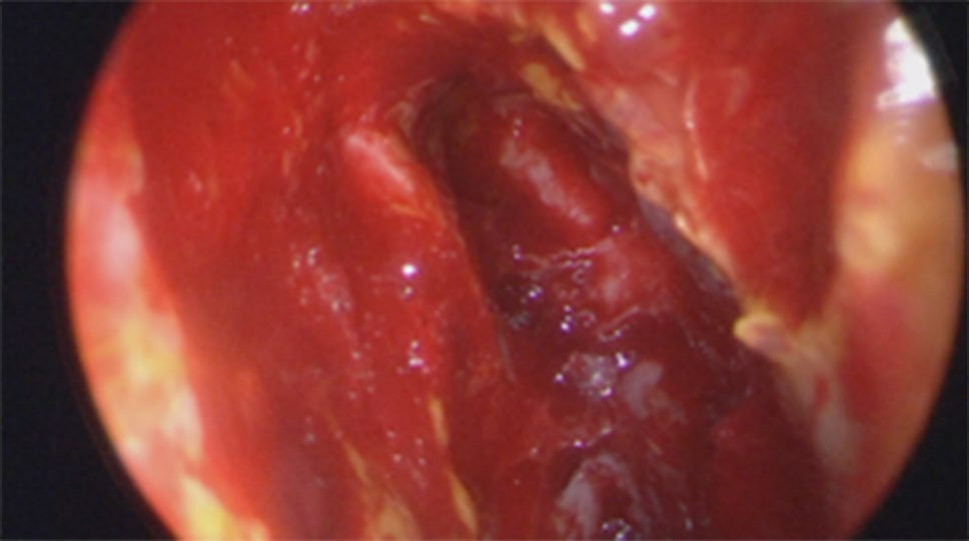
Frontal sinus cleared after rifocin injection

A Functional Endoscopic Sinus Surgery was conducted following the steps below:

Firstly we entered the right nasal nostril with a zero degree scope (Fig. 8) the mucosa of the nasal cavity appeared congested. The middle turbinate was then medialized (Fig. 9), allowing the identification of the posterior aspect of the uncinate process. The uncinate process was gently fractured with the angled probe. The maxillary sinus natural ostium was identified and a widened opening of the ostium was performed using a backbiting probe (Fig. 10). The maxillary antrum presented hyperplastic mucosa but no significant purulent discharge (Fig. 11). After verifying the lamina papyracea integrity, a complete antero-posterior ethmoidectomy was performed using a micro debrider to carefully fracture individual etmoidal septations (Fig. 12). A minimal purulent discharge was drained, probably a consequence of the frontal sinus purulent accumulation (Fig. 13). To reach the sphenoid sinus a transethmoidal approach was performed. (Fig. 14) The sphenoid sinus was clear with no purulent discharge (Fig. 15). For the primary identification of the frontal nasal sinus a curette was then used (Fig. 16). For the frontal nasal sinus approach a DRAF IIa was performed. A considerable amount of purulent fluid was drained (Fig. 17). The cavity was then washed using Rifocin solution (Figs.18, 19).

After the procedure the antibiotic was escalated to ceftriaxone (2 g 2 per day) with vancomicina (20 mg/kg 2 per day) plus metronidazole (500 mg × 4 per day). The next day a CT scan was obtained. It showed normal craniotomy outcome, with a small amount of remaining collection, and light enhancement after contrast administration. The results from the blood culture (on the admission) revealed Streptococcus constellatus. The culture of the purulent discharge from the ethmoidal cells was positive for Streptococcus constellatus and Staphylococcus epidermidis (multisensitive non tested for ceftriaxone). Culture from the frontal sinus revealed Streptococcus Constellatus and Parvimonas spp. The latter was also cultured on the material collected during craniotomy. On recovery after surgery the patient remained several days at the intensive care unit. For the first week the patient was difficult to arouse, only capable of following simple instructions, with no eye opening response. The patient also suffered from repetitive episodes of seizures only controllable with continuous infusion of midazolam. Approximately 1 week after admission the patient’s condition gradually improved. He regained full neurological function, was accompanied by a rehabilitation team on recovering power on the left extremities. At discharge the patient had no apparent neurological deficits.

## Discussion

Intracranial complications of pediatric rhino-sinusitis are rare in the post-antibiotic era, nonetheless they are potentially life threatening. These include meningitis, cavernous and sagittal sinus thrombosis, epidural or subdural empyema and cerebral abscesses. All these complications can lead to significant morbidity with permanent brain damage and sometimes to death. For this reason, early diagnosis and prompt treatment are essential.

Intracranial extension of acute sinusitis has a reported incidence of 3.7% to 11% in hospitalized patients [4]. The frontal sinuses are the most commonly associated with intracranial suppuration, followed in order by ethmoid, sphenoid and maxillary sinuses [5]. The incidence of intracranial complications is low in the young children and gets higher in the second decade. Epidemiological data strongly support the responsibility of the frontal sinus in most cases, since the development of the frontal sinuses is rarely complete before the age of ten [6, 7]. It is hypothesized that the frontal skeleton is more vulnerable to the spread of infections because of its abundant network of diploic veins (Veins of Breschet) [8] that interconnects the sinus mucosa to the meninges, skull and brain parenchyma.

Subdural empyemas are reported to be the most common complication, forming in 20% of complicated sinus disease before the age of 20. The intracranial spread of the infection is usually the result of retrograde thrombophlebitis of the diploic veins, while direct extension with osteomyelitis and erosion of the posterior wall of the frontal sinus is much less common [9]. In accordance with this epidemiological data, we report the case of a young adolescent male of 12 with a subdural empyema secondary to frontal sinusitis with intact walls of the frontal sinus.

The clinical presentation in these cases is often masked by prior antibiotic treatment, with initial mild and non-specific symptoms. Frontal headache and fever are the most common complaints, followed by fever, nausea and vomiting [10]. Less common signs and symptoms are neurological deficits, photophobia, lethargy, seizures and hemiparesis. In these cases, the evolution is very fast and a prompt surgical intervention is required. Our patient initially referred to mild symptoms with headache unresponsive to pain-relievers, however when he was brought in to the Emergency Department vomiting and photophobia were present. In a few hours, initial signs of hemiparesis occurred leading to the decision of an urgent surgical intervention.

Imaging study is key for the diagnosis. CT scan is usually the first investigation required. It clearly shows frontal sinus opacification and if signs of bone erosion are present. However, CT scans may fail in revealing intracranial complications. The American College of Radiology considers that MRI with contrast and contrast-enhanced CT scans are complementary examinations when evaluating potential complications of sinusitis [11]. MRI gadolinium-enhanced T1-weighted sequences are particularly useful in distinguishing epidural empyema from subdural empyema and in detecting meningeal enhancement.

Non-specific laboratory findings like neutrophilic leukocytosis are common. Blood cultures are negative in most cases, while pus samples often show a polymicrobial flora. A recent retrospective study found that Streptococcus anginosus group bacteria have been isolated in up to 77% rhinogenic intracranial infections. In our case, the results from the blood culture revealed Streptococcus constellatus, while intraoperative cultures of the purulent discharge from paranasal sinuses and the samples collected during craniotomy were positive for Streptococcus constellatus, Staphylococcus epidermidis and Parvimonas.

The treatment of sinogenic subdural empyema is based on neurosurgical intervention to remove the suppuration. Craniotomy with or without cranialization or exenteration of the frontal sinus is the treatment of choice. The decision to cranialize the frontal sinus is based on the involvement of the posterior table of the frontal sinus in the disease. When both the anterior and posterior tables are involved, frontal sinus exenteration is advisable [12]. In our case, since the tables of the frontal sinus were undamaged, only craniotomy with drainage of the empyema was performed. Functional endoscopic sinus surgery is usually associated in order to remove the sinus disease and to restore a correct ventilation of the paranasal cavities. FESS can be performed at the same time of the neurosurgery or it may be postponed. We decided to perform the two surgeries at the same time in order to abolish the source of the infection and to prevent a second anesthesia to the young patient. Medical treatment, while not being sufficient alone in treating the empyema, is always associated with the surgical intervention. Broad spectrum antibiotics should be used continuously for 4–8 weeks. The medical treatment can be modified with targeted antibiotics according to the result of the cultures.

## Conclusion

Subdural empyema is a rare but severe complication of pediatric sinusitis. The clinical picture may evolve in few hours, leading to significant morbidity with permanent brain damage and sometimes to death. For this reason, early diagnosis with combined medical and surgical therapies are recommended in order to reduce the risk of morbidity and mortality.

## Data Availability

Data sharing not applicable to this article as no datasets were generated or analysed during the current study.
